# Supplementary material for: Discovery of α-amylase and α-glucosidase dual inhibitors from NPASS database for management of Type 2 Diabetes Mellitus: A chemoinformatic approach
Source: PLoS One. 2024 Nov 14;19(11):e0313758. doi: 10.1371/journal.pone.0313758 (PMC11563405; doi:10.1371/journal.pone.0313758)
Supplement: S1 Fig — (DOCX) [file pone.0313758.s001.docx]

**Discovery of** α**-Amylase and** α**-Glucosidase Dual Inhibitors from NPASS Database for Management of Type 2 Diabetes: AI-Assisted Chemoinformatic Approach**


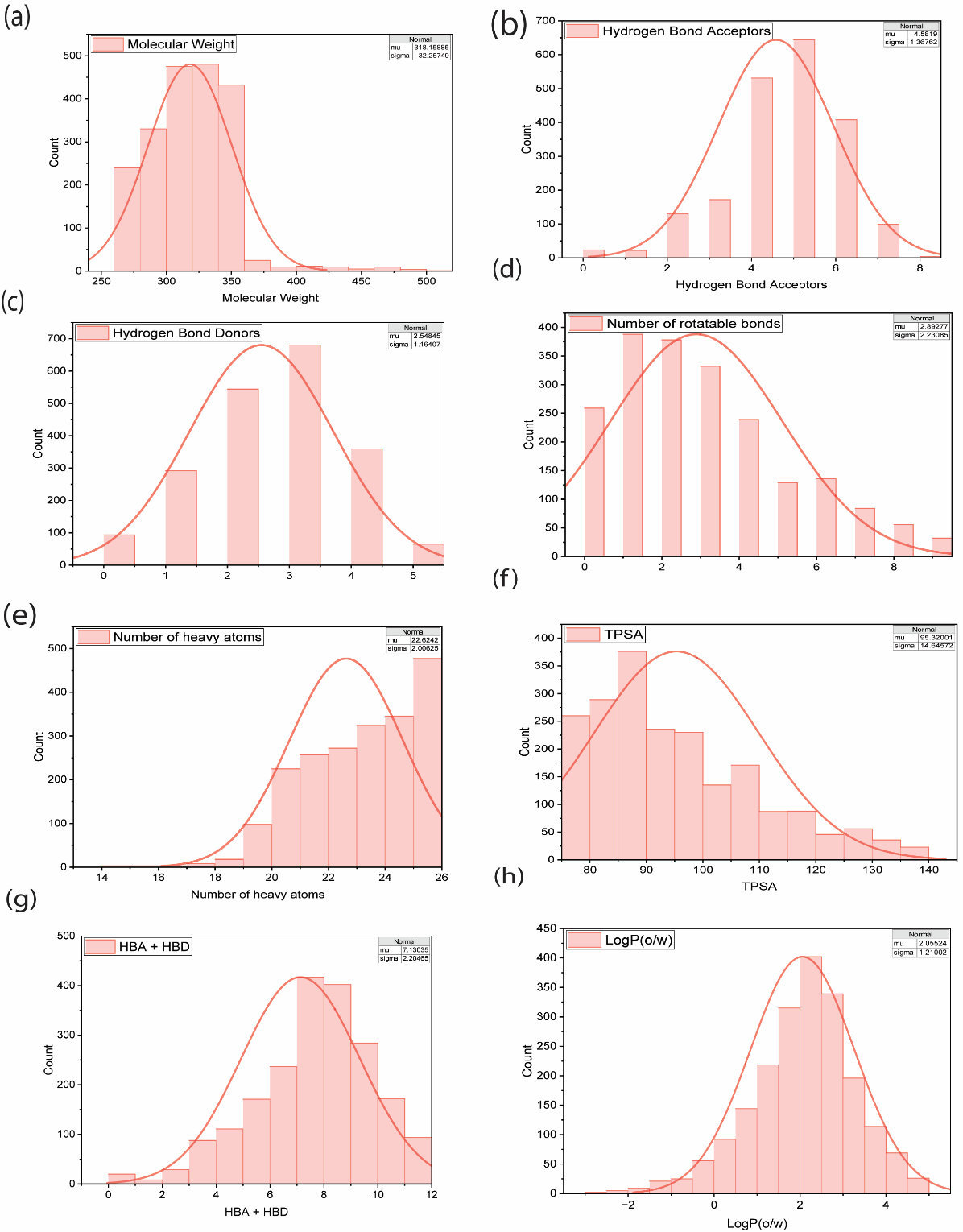


**S1** Fig 1:**Pharmacokinetics properties of the screened molecules**;(a) molecular weight, (b) Hydrogen bond acceptors, (c) Hydrogen bond donors, (d) Number of rotatable bonds, (e) Number of heavy atoms, (f) TPSA, (g) HBA +HBA, (h) LogP(o/w).
