# Supplementary material for: Discovery of α-amylase and α-glucosidase dual inhibitors from NPASS database for management of Type 2 Diabetes Mellitus: A chemoinformatic approach
Source: PLoS One. 2024 Nov 14;19(11):e0313758. doi: 10.1371/journal.pone.0313758 (PMC11563405; doi:10.1371/journal.pone.0313758)
Supplement: S1 Table — (DOCX) [file pone.0313758.s002.docx]

**S2** Table 1: **Docking score and the interactions of selected compounds and acarbose with the α-amylase residues discovered during structure visualization**.

| **Compound ID** | **Binding residues** | | **Interactions** | | | **Docking score** | **Rmsd** |
| --- | --- | --- | --- | --- | --- | --- | --- |
|  | *ligand* | *Receptor* | *Type of interaction* | *Distance* | *E(kcal/mol)* |  |  |
| NPC204580 | C 18  C 21  O 37  N 19  6-ring | OD1 ASP 300  OE1 GLU 233  NZ LYS 200  OD1 ASP 197  CE1 HIS 201 | H-donor  H-donor  H-acceptor  Ionic  pi-H | 3.25  3.64  3.50  3.60  3.48 | -1.5  -0.6  -1.1  -1.5  -0.9 | -14.4621 | 1.8020 |
| NPC137813 | O 34  O 35  O 34  O 35 | OD1 ASP 300  OD1 ASP 197  NE2 HIS 299  NH2 ARG 195 | H-donor  H-donor  H-acceptor  H-acceptor | 2.83  2.75  2.97  3.11 | -4.2  -4.2  -1.5  -0.4 | -12.5790 | 1.7879 |
| NPC76084 | O 33  O 37  O 33 | OD1 ASP 197  OE1 GLU 233  NE2 HIS 299 | H-donor  H-donor  H-acceptor | 2.89  2.72  3.37 | -3.1  -3.3  -1.2 | -11.5494 | 1.9626 |
| NPC27750 | O 6  O 7  O 7  O 22 | NH2 ARG 195  NH2 AGR 195  NE2 HIS 299  NE2 HIS 305 | H-acceptor  H-acceptor  H-acceptor  H-acceptor | 3.45  2.99  2.98  2.92 | -1.1  -2.3  -5.1  -3.2 | -10.1729 | 1.9334 |
| Acarbose | O 17  C 34 | OE1 GLN 63  6-ring TRP 59 | H-donor  H-pi | 2.97  4.13 | -1.1  -0.6 | -12.9946 | 1.6148 |
