# Supplementary material for: Discovery of α-amylase and α-glucosidase dual inhibitors from NPASS database for management of Type 2 Diabetes Mellitus: A chemoinformatic approach
Source: PLoS One. 2024 Nov 14;19(11):e0313758. doi: 10.1371/journal.pone.0313758 (PMC11563405; doi:10.1371/journal.pone.0313758)
Supplement: S2 Table — (DOCX) [file pone.0313758.s003.docx]

**S3** Table 2: **Docking score and the interactions of selected top four compounds and acarbose with the α-glucosidase receptor upon visualization**.

| **Compound ID** | **Binding residues** | | **Interactions** | | | **Docking score** | **Rmsd** |
| --- | --- | --- | --- | --- | --- | --- | --- |
|  | *ligand* | *Receptor* | *Type of interaction* | *Distance* | *E(kcal/mol)* |  |  |
| NPC25750 | O 7 | NE2 HIS 600 | H-acceptor | 3.33 | -2.3 | -9.0762 | 1.1439 |
| NPC137813 | O 33  O 37  6-ring | OG1 THR 205  NH2 ARG 202  CA THR 204 | H-acceptor  H-acceptor  Pi-H | 3.12  3.42  4.30 | -0.6  -1.6  -0.9 | -8.7610 | 1.8797 |
| NPC76084 | O 33 | NH2 ARG 202 | H-acceptor | 3.06 | -0.7 | -8.5586 | 1.9705 |
| NPC204580 | C 16  C 21  N 19  N 19  C 34 | SD MET 444  OD2 ASP 542  OD1 ASP 203  OD2 ASP 542  6-ring PHE575 | H-donor  H-donor  Ionic  Ionic  H-pi | 4.36  3.17  3.63  3.88  4.19 | -0.7  -0.8  -1.4  -0.8  -0.6 | -8.4181 | 1.7932 |
| Acarbose | O 6  O 10  O 13  C 28  C 31  O 10  O 13  N 19 | OD2 ASP 203  OD1 ASP 542  OD2 ASP 327  SD MET444  OD2 ASP 443  NH1 ARG 526  NE2 HIS 600  OD1 ASP 542 | H-donor  H-donor  H-donor  H-donor  H-donor  H-acceptor  H-acceptor  Ionic | 3.14  2.58  2.83  4.03  2.98  2.99  3.20  2.80 | -1.6  -3.0  -3.8  -1.2  -0.9  -2.8  -0.9  -5.9 | -8.2153 | 1.2913 |
