# Supplementary material for: Discovery of α-amylase and α-glucosidase dual inhibitors from NPASS database for management of Type 2 Diabetes Mellitus: A chemoinformatic approach
Source: PLoS One. 2024 Nov 14;19(11):e0313758. doi: 10.1371/journal.pone.0313758 (PMC11563405; doi:10.1371/journal.pone.0313758)
Supplement: S3 Table — (DOCX) [file pone.0313758.s004.docx]

**S4** Table 3: **Top ten docked poses of the selected molecules; NPC137813, NPC204580, and the reference molecules Acarbose in 3BAJ and 2QMJ receptors**.

| **Top ten poses of NPC137813 with** α**-glucosidase docking scores** | | | | | | | |  |  |
| --- | --- | --- | --- | --- | --- | --- | --- | --- | --- |
| mol | rseq | mseq | S | rmsd_refine | E_conf | E_place | E_score1 | E_refine | E_score2 |
| NPC137813 | 1 | 2 | -8.76103 | 1.88837 | 85.29571 | -83.3143 | -9.99738 | -36.6662 | -9.2534 |
| NPC137813 | 1 | 2 | -8.75338 | 2.335788 | 85.44517 | -77.0259 | -11.2897 | -36.0383 | -9.19404 |
| NPC137813 | 1 | 2 | -8.75286 | 2.307614 | 81.04989 | -79.7755 | -9.9185 | -32.9176 | -8.82857 |
| NPC137813 | 1 | 2 | -8.65404 | 1.879722 | 80.33741 | -56.6988 | -11.4196 | -34.169 | -8.76103 |
| NPC137813 | 1 | 2 | -8.6334 | 2.032482 | 81.6442 | -47.1956 | -9.97571 | -37.6139 | -8.75338 |
| NPC137813 | 1 | 2 | -8.57736 | 1.422926 | 87.25224 | -73.1741 | -10.1413 | -28.6268 | -8.57736 |
| NPC137813 | 1 | 2 | -8.48899 | 2.347135 | 81.07302 | -68.2018 | -10.3797 | -36.5783 | -8.48899 |
| NPC137813 | 1 | 2 | -8.47267 | 1.840335 | 86.29304 | -52.6786 | -9.93029 | -34.4236 | -8.47267 |
| NPC137813 | 1 | 2 | -8.4563 | 0.858618 | 77.3781 | -61.8478 | -10.586 | -30.1771 | -8.4563 |
| NPC137813 | 1 | 2 | -7.97356 | 3.661563 | 80.2841 | -72.1777 | -10.9016 | -36.5147 | -7.97356 |
|  |  |  |  |  |  |  |  |  |  |
| **Top ten poses of NPC204580 with α-glucosidase docking scores** | | | | | | | |  |  |
| mol | rseq | mseq | S | rmsd_refine | E_conf | E_place | E_score1 | E_refine | E_score2 |
| NPC204580 | 1 | 1 | -8.41812 | 1.81453 | 37.66348 | -74.4597 | -12.6435 | -30.7556 | -9.53172 |
| NPC204580 | 1 | 1 | -8.33172 | 1.414635 | 46.23306 | -72.5077 | -11.7797 | -27.0719 | -9.46326 |
| NPC204580 | 1 | 1 | -8.26326 | 2.281953 | 51.31535 | -95.1984 | -12.7989 | -26.1012 | -9.34617 |
| NPC204580 | 1 | 1 | -8.14617 | 1.64102 | 51.29466 | -30.0781 | -12.3616 | -26.0921 | -9.3434 |
| NPC204580 | 1 | 1 | -7.91415 | 2.313433 | 48.75798 | -59.6185 | -11.7639 | -21.648 | -9.00827 |
| NPC204580 | 1 | 1 | -7.90918 | 2.344837 | 33.95421 | -51.8933 | -13.8899 | -26.6873 | -8.9918 |
| NPC204580 | 1 | 1 | -7.8434 | 1.68587 | 48.60243 | -47.6315 | -11.436 | -18.739 | -8.95654 |
| NPC204580 | 1 | 1 | -7.55654 | 1.830331 | 37.8803 | -40.4604 | -11.6829 | -27.0826 | -8.89724 |
| NPC204580 | 1 | 1 | -7.00827 | 1.793209 | 46.58095 | -58.2082 | -11.7229 | -15.3986 | -8.41812 |
| NPC204580 | 1 | 1 | -6.99724 | 1.450214 | 51.88573 | -57.3436 | -12.235 | -6.6618 | -7.91415 |
|  |  |  |  |  |  |  |  |  |  |
| **Top ten poses of Acarbose (reference) with** α**-glucosidase docking scores** | | | | | | | | |  |
| mol | rseq | mseq | S | rmsd_refine | E_conf | E_place | E_score1 | E_refine | E_score2 |
| Acarbose | 1 | 1 | -8.21532 | 1.291318 | 212.3576 | -120.893 | -18.6244 | -51.7974 | -8.21532 |
| Acarbose | 1 | 1 | -8.20762 | 1.899994 | 209.3934 | -72.1205 | -7.54501 | -51.1153 | -8.20762 |
| Acarbose | 1 | 1 | -8.09206 | 2.439259 | 203.5718 | -53.2627 | -7.76199 | -49.3812 | -8.09206 |
| Acarbose | 1 | 1 | -7.85153 | 1.972781 | 208.2269 | -11.193 | -12.9505 | -50.7465 | -7.85153 |
| Acarbose | 1 | 1 | -7.61379 | 2.078672 | 202.6323 | -51.2126 | -10.1361 | -40.9282 | -7.61379 |
| Acarbose | 1 | 1 | -6.68334 | 3.878601 | 209.0457 | -22.5269 | -7.34448 | -30.6757 | -6.68334 |
| Acarbose | 1 | 1 | -6.53984 | 3.653367 | 211.0734 | -18.6017 | -8.35201 | -24.7315 | -6.53984 |
| Acarbose | 1 | 1 | -6.43713 | 3.176234 | 202.4566 | -15.052 | -7.37009 | -30.7312 | -6.43713 |
| Acarbose | 1 | 1 | -6.26708 | 2.066111 | 201.5151 | -31.8053 | -10.4304 | -26.3252 | -6.26708 |
| Acarbose | 1 | 1 | -6.18494 | 1.642416 | 195.9855 | -37.8749 | -9.1099 | -34.1819 | -6.18494 |
|  |  |  |  |  |  |  |  |  |  |
| **Top ten poses of NPC137813 with** α**-amylase docking scores** | | | | | | |  |  |  |
| mol | rseq | mseq | S | rmsd_refine | E_conf | E_place | E_score1 | E_refine | E_score2 |
| NPC137813 | 1 | 2 | -12.579 | 1.787946 | 82.39311 | -109.39 | -9.45829 | -46.6145 | -12.579 |
| NPC137813 | 1 | 2 | -12.4238 | 2.800655 | 83.07259 | -78.0329 | -11.381 | -48.1373 | -12.0238 |
| NPC137813 | 1 | 2 | -12.3167 | 2.212599 | 80.9527 | -73.6801 | -9.92959 | -43.9197 | -12.0167 |
| NPC137813 | 1 | 2 | -12.2597 | 2.020088 | 80.90101 | -81.0404 | -10.0047 | -47.8929 | -11.8597 |
| NPC137813 | 1 | 2 | -12.1003 | 2.068335 | 81.28394 | -57.3591 | -9.42862 | -41.4155 | -11.6003 |
| NPC137813 | 1 | 2 | -11.9654 | 1.796654 | 79.8654 | -96.8604 | -11.8691 | -38.8472 | -11.4654 |
| NPC137813 | 1 | 2 | -11.7918 | 1.808322 | 76.39182 | -51.2327 | -10.1116 | -42.3986 | -11.3918 |
| NPC137813 | 1 | 2 | -11.6813 | 1.276934 | 78.07275 | -73.426 | -11.099 | -37.4805 | -11.3813 |
| NPC137813 | 1 | 2 | -11.571 | 2.674014 | 79.4208 | -60.1333 | -9.64488 | -32.5571 | -11.271 |
| NPC137813 | 1 | 2 | -11.2295 | 1.821809 | 83.52885 | -72.2422 | -11.3819 | -37.622 | -11.2295 |
|  |  |  |  |  |  |  |  |  |  |
| **Top ten poses of NPC204580 with** α**-amylase docking scores** | | | | | | |  |  |  |
| mol | rseq | mseq | S | rmsd_refine | E_conf | E_place | E_score1 | E_refine | E_score2 |
| NPC204580 | 1 | 1 | -14.4621 | 1.802011 | 35.44961 | -77.4254 | -11.2925 | -51.3837 | -14.4621 |
| NPC204580 | 1 | 1 | -14.4618 | 1.764134 | 35.45652 | -85.0054 | -11.6847 | -51.3917 | -14.4618 |
| NPC204580 | 1 | 1 | -14.4515 | 1.555651 | 37.22322 | -51.1362 | -12.6259 | -52.3352 | -14.4515 |
| NPC204580 | 1 | 1 | -13.8753 | 1.501828 | 41.37893 | -65.137 | -12.082 | -41.9747 | -13.8753 |
| NPC204580 | 1 | 1 | -13.5826 | 1.381291 | 35.50892 | -62.686 | -12.1202 | -47.2696 | -13.5826 |
| NPC204580 | 1 | 1 | -13.4791 | 1.311957 | 31.43153 | -70.639 | -10.9814 | -43.5377 | -13.4791 |
| NPC204580 | 1 | 1 | -13.1947 | 2.065751 | 37.44419 | -70.5433 | -12.8651 | -46.7403 | -13.1947 |
| NPC204580 | 1 | 1 | -13.0065 | 2.262682 | 34.99592 | -70.1336 | -11.516 | -45.7864 | -13.0065 |
| NPC204580 | 1 | 1 | -12.6875 | 2.242865 | 37.04658 | -72.9694 | -10.949 | -45.9202 | -12.6875 |
| NPC204580 | 1 | 1 | -12.4081 | 1.318545 | 37.40218 | -75.272 | -11.4409 | -34.5008 | -12.4081 |
|  |  |  |  |  |  |  |  |  |  |
| **Top ten poses of Acarbose (reference) with** α**-amylase docking scores** | | | | | | | |  |  |
| mol | rseq | mseq | S | rmsd_refine | E_conf | E_place | E_score1 | E_refine | E_score2 |
| Acarbose | 1 | 1 | -12.9946 | 1.614768 | 215.7984 | -96.1983 | -12.287 | -32.0879 | -12.9946 |
| Acarbose | 1 | 1 | -12.939 | 2.287654 | 207.5058 | -58.3788 | -12.4294 | -38.9517 | -12.939 |
| Acarbose | 1 | 1 | -12.8094 | 1.62795 | 202.8693 | -78.2057 | -12.5704 | -56.5978 | -12.8094 |
| Acarbose | 1 | 1 | -12.7258 | 2.19905 | 210.229 | -63.6874 | -13.7739 | -42.27 | -12.7258 |
| Acarbose | 1 | 1 | -12.6237 | 1.378166 | 199.1647 | -97.7129 | -13.3631 | -49.572 | -12.6237 |
| Acarbose | 1 | 1 | -12.5692 | 2.531663 | 200.1203 | -86.9518 | -13.1016 | -56.4331 | -12.5692 |
| Acarbose | 1 | 1 | -12.3928 | 2.056788 | 197.6421 | -84.6215 | -12.4115 | -49.4384 | -12.3928 |
| Acarbose | 1 | 1 | -12.2175 | 1.401791 | 202.1732 | -101.743 | -16.0715 | -47.6254 | -12.2175 |
| Acarbose | 1 | 1 | -12.2072 | 0.771867 | 204.1883 | -96.3818 | -14.8308 | -47.1893 | -12.2072 |
|  | 1 | 1 | -12.0608 | 2.252975 | 201.2899 | -94.0029 | -12.5127 | -44.6178 | -12.0608 |
