# Supplementary material for: Discovery of α-amylase and α-glucosidase dual inhibitors from NPASS database for management of Type 2 Diabetes Mellitus: A chemoinformatic approach
Source: PLoS One. 2024 Nov 14;19(11):e0313758. doi: 10.1371/journal.pone.0313758 (PMC11563405; doi:10.1371/journal.pone.0313758)
Supplement: Attachment — Submitted filename: Response to Reviewers.docx [file pone.0313758.s005.docx]

**Discovery of α-Amylase and α-Glucosidase Dual Inhibitors from NPASS Database for Management of Type 2 Diabetes Mellitus: A Chemoinformatic Approach**

**Point-by-Point Response to Editor and Reviewers’ Comments**

| **Comments** | **Authors’ response/action** | **Text revised** |
| --- | --- | --- |
| **Editor** | | |
| Please ensure that your manuscript meets PLOS ONE's style requirements, including those for file naming. The PLOS ONE style templates can be found at  <https://journals.plos.org/plosone/s/file?id=wjVg/PLOSOne_formatting_sample_main_body.pdf> and  <https://journals.plos.org/plosone/s/file?id=ba62/PLOSOne_formatting_sample_title_authors_affiliations.pdf> | We appreciate the comment and observation. The guidelines have been adhered to. | Text |
| Please note that funding information should not appear in any section or other areas of your manuscript. We will only publish funding information present in the Funding Statement section of the online submission form. Please remove any funding-related text from the manuscript. | This has been addressed | Text |
| We note that your Data Availability Statement is currently as follows: All relevant data are within the manuscript and its Supporting Information files.  Please confirm at this time whether or not your submission contains all raw data required to replicate the results of your study. Authors must share the “minimal data set” for their submission. PLOS defines the minimal data set to consist of the data required to replicate all study findings reported in the article, as well as related metadata and methods (<https://journals.plos.org/plosone/s/data-availability#loc-minimal-data-set-definition>).  For example, authors should submit the following data:  - The values behind the means, standard deviations and other measures reported; - The values used to build graphs; - The points extracted from images for analysis.  Authors do not need to submit their entire data set if only a portion of the data was used in the reported study.  If your submission does not contain these data, please either upload them as Supporting Information files or deposit them to a stable, public repository and provide us with the relevant URLs, DOIs, or accession numbers. For a list of recommended repositories, please see <https://journals.plos.org/plosone/s/recommended-repositories>.  If there are ethical or legal restrictions on sharing a de-identified data set, please explain them in detail (e.g., data contain potentially sensitive information, data are owned by a third-party organization, etc.) and who has imposed them (e.g., an ethics committee). Please also provide contact information for a data access committee, ethics committee, or other institutional body to which data requests may be sent. If data are owned by a third party, please indicate how others may request data access. | We confirm that our manuscript contains all the relevant data within the manuscript and its supporting information file. | Text |
| Please include captions for your Supporting Information files at the end of your manuscript, and update any in-text citations to match accordingly. Please see our Supporting Information guidelines for more information: <http://journals.plos.org/plosone/s/supporting-information>. | This has been addressed | Supporting information |
| Please review your reference list to ensure that it is complete and correct. If you have cited papers that have been retracted, please include the rationale for doing so in the manuscript text, or remove these references and replace them with relevant current references. Any changes to the reference list should be mentioned in the rebuttal letter that accompanies your revised manuscript. If you need to cite a retracted article, indicate the article’s retracted status in the References list and also include a citation and full reference for the retraction notice. | We have thoroughly reviewed our references list to ensure that it is both complete and correct. | References |
| **Reviewer # 1** | | |
| Binding Score: The binding score may be presented as a line graph to show the scores of the to 10 or 20 poses from the docking. A single value of binding score (top pose) is not representative of the docking result. | Thank you for your valuable suggestion regarding the presentation of the binding scores. In response, we have included line graphs in the revised manuscript that show the binding scores of the top ten poses for each of the selected molecules. These graphs provide a more comprehensive view of the docking results and better represent the variation in binding affinities across the different poses, as you recommended.  We appreciate your feedback, and we believe the inclusion of these graphs will enhance the clarity of the docking results. Thank you once again for your insightful comments. | Fig 7 |
| ADMET and Drug-likeness screening: In light of the fact that the target enzymes are within the intestine, should high absorption be a consideration? Acarbose, the reference compound, is not well absorbed. | Thank you for your insightful comment regarding ADMET and drug-likeness screening. Given this, high absorption may not be a critical parameter for these molecules.  However, since the selected molecules are substrates of P-glycoprotein (P-gp), we anticipate that they will be effluxed back into the gastrointestinal tract. This efflux mechanism could localize their activity within the intestinal lumen, similar to acarbose, while minimizing systemic absorption. This localization would reduce the risk of off-target effects and enhance their safety profiles, aligning well with the therapeutic objectives for treating type 2 diabetes mellitus (T2DM).  We have clarified this point in the revised manuscript. | Results and discussion (Text) |
| The MD should include the reference compound, acarbose, for good comparison. | Thank you for your comment regarding the inclusion of acarbose in the molecular dynamics (MD) simulation for comparison. The primary aim of the MD simulation in our study was to assess the stability and binding free energy of the docked ligands within the active sites of the two target proteins, without focusing on direct comparison with reference molecules.  Moreover, the exclusion of acarbose from the MD simulation was due to considerations related to computational cost and the availability of necessary resources for running additional simulations. While we acknowledge the value of including a reference for comparison, our focus was on thoroughly evaluating the behavior of the selected ligands in the active site. | Text |
| **Reviewer # 2** | | |
| Manuscript Title: Discovery of α-Amylase and α-Glucosidase Dual Inhibitors from NPASS Database for Management of Type 2 Diabetes: A Chemoinformatic Approach  Manuscript Number: PONE-D-24-24347 Authors: Wilberforce Ndarawit, Charles Otieno Ochieng, David Angwenyi, Jorddy N. Cruz, Cleydson B.R. Santos, Njogu M. Kimani, Ph.D Journal: PLOS ONE  Summary The manuscript titled "Discovery of α-Amylase and α-Glucosidase Dual Inhibitors from NPASS Database for Management of Type 2 Diabetes: A Chemoinformatic Approach" presents a study focused on the identification of potential dual inhibitors for α-amylase and α-glucosidase enzymes, which are relevant in the management of Type 2 Diabetes Mellitus (T2DM). The research utilizes computer-aided drug design (CADD) techniques to screen the Natural Products Activity and Species Source (NPASS) database, identifying two promising compounds: NPC204580 (Chrotacumine C) and NPC137813 (1-O-(2-Methoxy-4-Acetylphenyl)-6-O-(E-Cinnamoyl)-Beta-D-Glucopyranoside).  Evaluation Originality and Significance: The manuscript addresses a significant health issue, T2DM, by proposing a novel approach to identify dual enzyme inhibitors using chemoinformatic techniques. The application of CADD to screen a large natural product database is innovative and demonstrates a modern approach to drug discovery. Methodology: The authors employ a rigorous and well-structured methodology, including virtual screening, molecular docking, and molecular dynamic simulations, to identify and validate potential inhibitors. The study effectively combines various computational techniques to evaluate drug-likeness, pharmacokinetics, and toxicity profiles of the identified compounds. Results: The identification of NPC204580 and NPC137813 as potential dual inhibitors is well-supported by the computational data presented. The binding affinities, stability assessments, and pharmacokinetic properties of these compounds suggest their potential efficacy in managing postprandial hyperglycemia. Discussion and Conclusion:  The discussion is comprehensive, addressing the significance of the findings and comparing them with existing inhibitors. The conclusion appropriately highlights the need for further in vitro and in vivo studies to validate the therapeutic potential of the identified compounds. Presentation:  The manuscript is well-organized, with clear sections and logical flow. Figures and tables are appropriately used to illustrate the computational results and support the conclusions. Recommendation Accept Without Corrections  The manuscript is well-written and presents a thorough and innovative study. The findings are significant and contribute valuable insights into the discovery of dual enzyme inhibitors for the management of T2DM. I recommend accepting the manuscript without any corrections. | Thank you for your detailed and encouraging evaluation of our manuscript. We are glad you found our study *on in-silico* identification of dual enzyme inhibitors for management P. hyperglycemia to be significant and innovative. Your recognition of our methodology, results, and overall presentation strengthens our commitment to advancing this research. We appreciate your recommendation for acceptance without correction. | Text |
|  |  |  |
| **Reviewer # 3** | | |
| The manuscript Discovery of α-Amylase and α-Glucosidase Dual Inhibitors from NPASS Database for Management of Type 2 Diabetes: A Chemoinformatic Approach. by Ndarawit.et al is about the computer-aided drug design (CADD). Find below section wise comments Title: Title of the work is attractive and informative, needs no change. | Thank you for your positive feedback on the title of our manuscript. | Title |
| Abstract: The work carried out by the authors are very much impressive, results obtained are quite interesting but unfortunately the work is not reflected from the abstract, I suggest authors to rewrite the abstract to make it clearer and more self-explanatory. Following un-necessary statements should be deleted from the abstract section "Postprandial hyperglycemia, typical manifestation of Type 2 Diabetes Mellitus (T2DM) characterized by elevated blood sugar levels, is associated with notable global morbidity and mortality. Preventing the advancement of this condition by delaying the rate of glucose absorption through inhibition of α-amylase and α-glucosidase enzymatic activities is of utmost importance. Finding a safe antidiabetic drug is essential since the one that are currently on the market have drawbacks like unpleasant side effects. Consequently, " | Thank you for your thoughtful feedback on the abstract. We have carefully considered your suggestions and made revisions to streamline the abstract. While some of the suggested deletions have been implemented, we have retained key sections that, in our view, are crucial to building the context for our research. These portions provide a necessary background to emphasize the significance of our work in addressing the challenges of T2DM. We hope this strikes a balance between clarity and providing a comprehensive overview of our study. | Abstract |
| Language: The overall quality of the language is fine, however, there are some minor typo/syntax errors in introduction and in results Discussion section, the authors should carefully go through the manuscript and correct the language issues. | We appreciate your observation and we have reviewed the introduction and the results and discussion sections correcting any typos or syntax error in the revised manuscript. | Text |
| Introduction: This section is very well written, well managed and well organized, however the authors should use relevant and recent literature/citations to make the manuscript more attractive, after careful reading I suggest the following mandatory replacements Replace reference number 3, 4, 5, 7 and 8 with <https://doi.org/10.3389/fendo.2023.1276836> doi: <https://doi.org/10.1186/s12951-022-01715-z> doi: <https://doi.org/10.1016/j.ejphar.2024.176356> doi: 10.1007/s00125-023-05992-7 doi: 10.1039/D3FO05456J with the mentioned minor mandatory points, I recommend this manuscript for publication. | Thank you for your thoughtful suggestions regarding the citations. After reviewing the suggested references, we carefully considered their relevance to our work. While they are valuable, we decided to retain current citations as they are more closely aligned with the specific context and scope of our study. The existing reference better supports the foundational aspects of the research. we appreciate your understanding and recommendation for publication. | Introduction |
| **Reviewer # 4** | | |
| The authors have conducted an interesting work which has good impact on field. The authors represented the work well and the results have been shown very clearly. I recommend it publication and to undergo the wet lab analysis to strengthen these findings. | Thank you for your encouraging feedback and recommendation for publication. We are pleased that you found our work interesting and impactful. We acknowledge that conducting wet lab analysis of the proposed molecules will strengthen our findings, and we are committed to pursuing this in our next step in our research. | Text |
